# Factors associated with unintended pregnancy in Brazil: cross-sectional results from the Birth in Brazil National Survey, 2011/2012

**DOI:** 10.1186/s12978-016-0227-8

**Published:** 2016-10-17

**Authors:** Mariza Miranda Theme-Filha, Marcia Leonardi Baldisserotto, Ana Claudia Santos Amaral Fraga, Susan Ayers, Silvana Granado Nogueira da Gama, Maria do Carmo Leal

**Affiliations:** 1 Department of Epidemiology and Quantitative Methods on Health, National School of Public Health, Oswaldo Cruz Foundation, Rio de Janeiro, Brazil; 2 Epidemiology in Public Health, Auxiliar Researcher National School of Public Health, Oswaldo Cruz Foundation, Rio de Janeiro, Brazil; 3 Epidemiology in Public Health, National Institute of Infectious Disease, Oswaldo Cruz Foundation, Rio de Janeiro, Brazil; 4 Psychology, Centre for Maternal and Child Health Research, City University, London, UK

**Keywords:** Unintended pregnancy, Unplanned pregnancy, Risk factors, Epidemiology, Brazil

## Abstract

**Background:**

Unintended pregnancy, a pregnancy that have been either unwanted or mistimed, is a serious public health issue in Brazil. It is reported for more than half of women who gave birth in the country, but the characteristics of women who conceive unintentionally are rarely documented. The aim of this study is to analyse the prevalence and the association between unintended pregnancy and a set of sociodemographic characteristics, individual-level variables and history of obstetric outcomes.

**Methods:**

Birth in Brazil is a cross-sectional study with countrywide representation that interviewed 23,894 women after birth. The information about intendedness of pregnancy was obtained after birth at the hospital and classified into three categories: intended, mistimed or unwanted. Multinomial regression analysis was used to estimate the associations between intendedness of a pregnancy, and sociodemographic and obstetric variables, calculating odds ratios and 95 % confidence intervals. All significant variables in the bivariate analysis were included in the multinomial multivariate model and the final model retaining variables that remained significant at the 5 % level.

**Results:**

Unintended pregnancy was reported by 55.4 % of postpartum women. The following variables maintained positive and significant statistical associations with mistimed pregnancy: maternal age < 20 years (OR = 1.89, 95 % CI: 1.68–2.14); brown (OR = 1.15, 95 % CI: 1.04–1.27) or yellow skin color (OR = 1.56, 95 % CI: 1.05–2.32); having no partner (OR = 2.32, 95 % CI: 1.99–2.71); having no paid job (OR = 1.15, 95 % CI: 1.04–1.27); alcohol abuse with risk of alcoholism (OR = 1.25, 95 % CI: 1.04–1.50) and having had three or more births (OR = 2.01, 95 % CI: 1.63–2.47). The same factors were associated with unwanted pregnancy, though the strength of the associations was generally stronger. Women with three or more births were 14 times more likely to have an unwanted pregnancy, and complication in the previous pregnancies and preterm birth were 40 % and 19 % higher, respectively. Previous neonatal death was a protective factor for both mistimed (OR = 0.61, 95 % CI: 0.44–0.85) and unwanted pregnancy (OR = 0.44, 95 % CI: 0.34–0.57).

**Conclusions:**

This study confirms findings from previous research about the influence of socioeconomic and individual risk factors on unintended pregnancy. It takes a new approach to the problem by showing the importance of previous neonatal death, preterm birth and complication during pregnancy as risk factors for unintended pregnancy.

**Electronic supplementary material:**

The online version of this article (doi:10.1186/s12978-016-0227-8) contains supplementary material, which is available to authorized users.

## Background

For a range of social and economic reasons, most individuals and couples want to plan the timing and spacing of their childbearing and avoid unintended pregnancy. Moreover, unintended pregnancy, defined as a pregnancy that is not desired at that particular time (mistimed) or not wanted at all (unwanted) [1], is a health problem in developed and middle- and low-income countries [2, 3]. The worldwide rate of unintended pregnancy in 2012 was 53 per 1000 women aged 15–44. The highest regional rate was in Africa (80 per 1000), and the lowest were in Europe and Oceania (43 per 1000) [4]. Almost half of all pregnancies in the United States are mistimed or unwanted [5].

Many risk factors have been identified for unintended pregnancy. The literature suggests socioeconomic disadvantage, lack of social support, maternal age, parity and maternal behaviours such as smoking and alcohol consumption are the strongest risk factors [6, 7]. These risk factors appear to be stable across cultures. A frequent consequence of unintended pregnancy is induced abortion, which is often unsafe in countries where the practice is illegal. Furthermore, births resulting from unintended pregnancy present more adverse maternal and child health outcomes such as delayed prenatal care, premature birth and negative physical and mental health effects [8–10]. All these factors increase health costs for neonatal care and costs associated with long-term disabilities for women and babies [11, 12].

In Brazil, unintended pregnancy remains high despite a dramatic reduction in fertility rates in the last decades, achieving 1.8 births per woman in 2011 [13], and the widespread use of contraceptive methods. According to the last Brazilian Demographic and Health Survey, performed in 2006, 67.8 % of women who had sex in the last 12 months were using some type of method of modern contraception. The same research showed that 53.9 % of all births in the last 5 years were unintended [14]. In Brazil, elective abortion is legal only when the pregnancy resulted from a rape, would cause a life-threatening condition to the mother, or the foetus has anencephaly or any other malformation that is incompatible with extrauterine life. As a result, the rate of unintended pregnancies that are carried to parturition is high [11].

Considering the importance of a more comprehensive analysis of the intendedness of a pregnancy for the health of women and their children, the objective of this study was to analyse the association between mistimed or unwanted pregnancies and sociodemographic characteristics, individual factors and history of previous obstetric risk factors among women who gave birth in hospitals included in the Birth in Brazil National Survey from 2011 to 2012.

## Methods

### Sample and study design

Birth in Brazil is an investigation with countrywide representation that interviewed 23,894 women after they gave birth, as well as collecting data from the mother’s and baby’s medical records, from February 2011 to October 2012. The sample was selected in three stages. First, hospitals that had 500 or more births in 2007 were selected. These were classified according to Brazil’s five macro-regions (north, northeast, southeast, south and mid-west), location (capital or non-capital), and type of hospital (private, public and mixed). Subsequently, the number of days needed to reach the fixed sample size of 90 women who had recently given birth (minimum of 7 days in each hospital) was calculated. The last stage was the selection of 90 women who had recently given birth from each hospital in the sample. The sample was distributed over 266 hospitals in 191 municipalities, including all state capitals. We considered eligible all postpartum women with hospital birth, having as its outcome a live birth, regardless of weight or gestational age, or stillbirth, weighing more than 500 g or gestational age greater than 22 weeks.

In the study’s first phase, face-to-face interviews were conducted with the women during hospitalization, data were taken from the mothers’ and children’s medical records and the women’s prenatal cards were photographed. Electronic forms were developed and validated to collect data and all interviews were conducted by interviewers previously trained by the investigation coordinators. Field research supervisors reapplied the questionnaire to a random sample of 5 % in the interviews with the women. Manuals were prepared with descriptions of procedures for data collection in order to ensure the quality of data and thereby minimize systematic errors.

As this was a complex sample, a calibration procedure was used, along with sample weights to ensure coherence between the sample estimates and known population totals obtained by an external source. Further details about sampling can be found in Vasconcellos et al. [15].

### Study variables

The outcome variable was pregnancy intention at conception. The women were asked whether their pregnancy was wanted at the time they became pregnant, with three possibilities: the pregnancy was intended (planned or at right time), mistimed (wanted later) or unwanted (never wanted).

The following sets of variables were included in the study to test their associations between mistimed and unwanted pregnancy:

- Sociodemographic variables: maternal age at delivery (<20; 20–34; ≥ 35); skin color (white, black, brown and yellow); and years of schooling (≤7; 8–11; ≥ 12). The skin color categories were based on Brazilian Demographic Census classification.
- Individual variables: marital status (with or without partner); having paid work; alcohol and tobacco use; and parity (primiparous, one child, two children and three or more children).
- Adverse obstetric outcomes in previous pregnancies: neonatal death; stillbirth; low birth weight (birth weight < 2500 g); preterm birth (gestational age < 37 weeks) and serious complications during pregnancy (eclampsia, diabetes, high blood pressure or uterine rupture).

The individual-level variable of alcohol use was measured using the TWEAK instrument, which was originally developed to identify habitual alcohol use among pregnant women [16]. Women who attained a score of at least three were considered at risk of alcoholism. The variable was divided into three categories: did not ingest alcoholic beverages during pregnancy, ingested alcoholic beverages but no alcoholism risk exists and ingested alcoholic beverages with risk of alcoholism. Women were considered smokers if they had smoked at least one cigarette per day during their pregnancy.

### Data analysis

Initially the characteristics of the women according to intendedness of pregnancy (intended, mistimed and unwanted) were described. Subsequently, to estimate the associations between mistimed and unwanted pregnancy with the study’s variables, a bivariate analysis using multinomial regression was performed. Odds ratios (OR) and 95 % confidence intervals were calculated, and intended pregnancy taken as the reference category. All significant variables in the bivariate analysis were included in the multinomial multivariate analysis, using hierarchical model. For each block of variables (sociodemographic, individual and obstetric variables), the significant variables from the previous block and the same block were controlled, with the final model retaining the variables that remained significant at the 5 % level.

The analyses were performed using SPSS 17.0 (Chicago, IL, USA), and because this was a complex sample, the Complex Sample was used to correct for design effects. This study followed the STROBE (Strengthening the Reporting of Observational Studies in Epidemiology) recommendations for the reporting of cross-sectional research [17].

### Ethical aspects

The hospital interview was performed following the signing of a free and informed consent form, which included authorization for subsequent telephone contact. The Birth in Brazil research was approved by the Sérgio Arouca National Public Health School Ethics Committee (CAAE 0096.0.031.000-10).

## Results

In total, 23,894 women were interviewed. The prevalence of unintended pregnancy in this study was 55.4 % of postpartum women. The percentages of pregnancies that were mistimed or unwanted were 25.5 % and 29.9 %, respectively.

Table 1 shows sociodemographic characteristics, individual variables and obstetric risk factors for the total sample and for the three groups analysed: intended, mistimed and unwanted pregnancy. The majority of the women interviewed were aged 20 to 34 years (70.4 %), of brown skin color (54.9 %), had 12 or more years of schooling (39 %), lived with a partner (81.4 %), had no paid work (59.7 %) and were primiparous (41.5 %). Regarding harmful behaviours, alcohol abuse and tobacco use were reported by 10 % and 9.6 % of the sample, respectively.

**Table 1:** Intendedness of births by sociodemographic, individual and obstetric history variables. Birth in Brazil, 2011/2012

Variable	Total		Intended	Mistimed	Unwanted
	N	%	%	%	%
Sociodemographic variables					
Maternal age (years)					
< 20	4571	19.1	34.7	33.5	31.8
20 to 34	16807	70.4	46.2	25.3	28.6
35 and over	2509	10.5	52.0	12.8	35.2
Schooling level					
≤ 7 years	6017	26.6	39.6	21.4	39.1
8 to 11 years	12537	25.6	44.6	26.5	28.9
12 or more years	5472	39.0	59.3	24.8	16.0
Skin color					
White	8701	36.2	52.7	23.8	23.5
Black	1879	7.8	40.8	23.1	36.1
Brown	13191	54.9	43.7	25.6	30.7
Yellow	265	1.1	41.3	29.9	28.8
Individual variables					
Marital status					
With partner	19440	81.4	49.5	25.0	25.5
Without partner	4432	18.6	22.9	28.0	49.1
Has paid work					
No	14272	59.7	40.5	26.6	32.8
Yes	9616	40.3	50.5	23.9	25.6
Alcohol use					
No	20070	86.0	46.2	25.5	28.3
Yes, but with no alcoholism risk	922	4.0	43.1	24.8	32.2
Yes, with alcoholism risk	2340	10.0	34.3	26.4	39.3
Tobacco use					
No	21579	90.4	45.7	26.2	28.1
Yes	2296	9.6	33.7	19.5	46.8
Parity					
Primiparous	9910	41.5	51.5	29.4	19.1
1	6697	28.0	46.6	26.0	27.5
2	3702	15.5	33.0	19.1	47.9
3 or more	3575	15.0	22.5	14.9	62.6
Adverse obstetric outcomes in previous pregnancy^a^					
Neonatal death					
No	12254	96.6	38.1	22.2	39.7
Yes	432	3.4	50.3	17.8	31.9
Stillbirth					
No	12129	95.6	35.7	16.6	32.7
Yes	558	4.4	41.3	20.5	38.3
Preterm birth (GA < 37 weeks)					
No	11176	88.1	39.2	22.4	38.4
Yes	1510	11.9	33.2	19.9	46.9
Low birth weight (BW < 2500 g)					
No	11039	87.0	39.1	22.6	38.4
Yes	1648	13.0	34.5	19.0	46.5
Maternal complications^b^					
No	10774	86.3	38.7	22.5	38.8
Yes	1717	13.7	36.2	19.8	44.0

*GA* gestational age, *BW* birth weight

^a^Excluding primiparous women

^b^Complications in previous pregnancy (eclampsia, high blood pressure, uterine rupture or diabetes)

In relation to obstetric history (excluding the current pregnancy and only for the multiparous), the most frequent events were preterm birth (11.9 %), low birth weight (13.0 %) and serious complications during pregnancy (13.7 %). Foetal loss and neonatal death was reported by 4.4 % and 3.4 % of mothers, respectively. It is important to note that these results refer to pregnancies carried to parturition, as all women were interviewed after delivery. Interruptions of unplanned pregnancies by abortion were not captured by the survey.

A very distinct profile can be observed when comparing the women who wanted to be pregnant at that time and those reporting a mistimed or unwanted pregnancy. Most women aged ≥ 35 years (52 %) wanted to be pregnant at that time, rather than reporting a mistimed or unwanted pregnancy. Relatively high percentages of those reporting white skin color (52.7 %), high educational level (59.3 % with 12 or more years of schooling), having a partner (49.5 %) and being primiparous (51.5 %) also stated that their pregnancy was intended. However, a high proportion of harmful behaviours, such as risk of alcoholism and tobacco use was observed in all groups, showing the strong association between these habits among women who did not planned the pregnancy. In relation to adverse outcomes in the previous pregnancies among the multiparous women, those who experienced previous neonatal death and stillbirth were more likely to report their pregnancy was intended. Women who experienced preterm birth, low birth weight and maternal complications were more likely to report unwanted pregnancy.

Table 2 presents the results of the bivariate multinomial regression analysis. All categories of sociodemographic and individual variables showed associations with unintended pregnancy and were tested in the multinomial multivariate model. Mistimed or unwanted pregnancy had inverse and significant associations with history of neonatal mortality (OR = 0.61, 95 % CI: 0.45–0.81 and OR = 0.61, 95 % CI: 0.48–0.76, respectively). Unwanted pregnancy had positive and significant associations with a history of low birth weight (OR = 1.37, 95 % CI: 1.18–1.59), preterm birth (OR = 1.44, 95 % CI 1.23–1.69) and complications in previous pregnancies (OR = 1.21, 95 % CI: 1.04–1.41).

**Table 2:** Unadjusted associations between study variables and mistimed and unwanted pregnancy. Birth in Brazil, 2011/2012

Variable	Mistimed (^a^)		Unwanted (^a^)	
	OR	95 % CI	OR	95 % CI
Sociodemographic variables				
Maternal age (years)				
< 20	1.76	1.57–1.98	1.48	1.32–1.67
35 and over	0.45	0.39–0.52	1.09	0.97–1.24
20 to 34	1	-	1	-
Schooling level				
≤ 7 years	1.21	1.05–1.39	3.37	2.94–3.88
8 to 11 years	1.32	1.16–1.50	2.29	2.01–2.62
12 or more years	1		1	
Skin color				
White	1	-	1	-
Black	1.19	1.00–1.42	1.75	1.49–2.06
Brown	1.26	1.15–1.40	1.49	1.35–1.65
Yellow	1.66	1.14–2.42	1.67	1.18–2.32
Individual variables				
Marital status				
With partner	1	-	1	-
Without partner	2.42	2.04–2.87	4.17	3.59–4.85
Has paid work				
Yes	1	-	1	-
No	1.39	1.26–1.53	1.60	1.46–1.75
Alcohol use				
No	1	-	1	-
Yes, but with no alcoholism risk	1.04	0.83–1.31	1.22	0.98–1.52
Yes, with alcoholism risk	1.40	1.17–1.67	1.87	1.64–2.14
Tobacco use				
No	1	-	1	-
Yes	1.01	0.86–1.18	2.26	1.98–2.57
Parity				
Primiparous	1	-	1	-
1	0.97	0.87–1.09	1.59	1.41–1.80
2	1.02	0.88–1.16	3.92	3.34–4.60
3 or more	1.16	0.96–1.40	7.51	6.35–8.88
Adverse obstetric outcomes in previous pregnancy^b^				
Neonatal death				
No	1		1	
Yes	0.61	0.46–0.81	0.61	0.48–0.76
Stillbirth				
No	1		1	
Yes	0.86	0.63–1.16	0.90	0.69–1.17
Preterm birth (GA < 37 weeks)				
No	1		1	
Yes	1.05	0.80–1.29	1.44	1.23–1.69
Low birth weight (BW < 2500 g)				
No	1		1	
Yes	0.95	0.80–1.14	1.37	1.18–1.59
Maternal complications^b^				
No	1		1	
Yes	0.95	0.81–1.12	1.22	1.04–1.42

*GA* gestational age, *BW* birth weight

(^a^) Reference category: intended pregnancy

^b^Excluding primiparous women

^c^Complications in previous pregnancy (eclampsia, high blood pressure, uterine rupture or diabetes)

The multivariate analysis adjusted for each block of variables (sociodemographic, individual and, for the multiparous, obstetric history) confirmed the results of the bivariate analysis. All variables that maintained a significant association with at least one category under analysis for mistimed or unwanted pregnancy were entered into the final multivariate model to identify key risk factors for unintended pregnancy in Brazil. Only stillbirth lost significance after adjustment for other variables.

The final model is presented in Table 3. The following variables maintained positive and significant statistical associations with mistimed pregnancy: maternal age under 20 years (OR = 1.89, 95 % CI: 1.68–2.14); being of brown (OR = 1.15, 95 % CI: 1.04–1.27) or yellow skin color (OR = 1.56, 95 % CI: 1.05–2.32); having no partner (OR = 2.32, 95 % CI: 1.99–2.71); having no paid job (OR = 1.15, 95 % CI: 1.04–1.27); alcohol abuse with risk of alcoholism (OR = 1.25, 95 % CI: 1.04–1.50) and parity. The larger the number of children, the higher the chance of a mistimed pregnancy.

**Table 3:** Final adjusted multinomial multivariate analysis predicting mistimed and unwanted pregnancy. Birth in Brazil, 2011/2012

Variables	Mistimed (^a^)		Unwanted (^a^)	
	OR	95 % CI	OR	95 % CI
Maternal Age (years)				
< 20	1.89	1.68–2.14	2.42	2.10–2.79
35 and over	0.43	0.37–0.51	0.75	0.64–0.88
20 to 34	1		1	
Schooling level				
≤ 7 years	0.72	0.63–0.83	1.04	0.89–1.21
8 to 11 years	0.99	0.87–1.12	1.38	1.21–1.58
12 or more years	1		1	
Skin color				
White	1		1	
Black	1.10	0.91–1.32	1.34	1.16–1.56
Brown	1.15	1.04–1.27	1.20	1.08–1.34
Yellow	1.56	1.05–2.32	1.39	0.93–2.08
Marital status				
With partner	1		1	
Without partner	2.32	1.99–2.71	4.86	4.12–5.74
Has paid work				
Yes	1		1	
No	1.15	1.04–1.27	1.14	1.04–1.25
Alcohol use				
No	1		1	
Yes, but with no alcoholism risk	1.12	0.90–1.39	1.21	0.96–1.53
Yes, with alcoholism risk	1.25	1.04–1.50	1.36	1.17–1.58
Tobacco use				
No	1			
Yes	0.87	0.73–1.04	1.23	1.06–1.43
Parity				
Primiparous	1			
1	1.30	1.16–1.46	2.49	2.15–2.88
2	1.55	1.34–1.80	6.96	5.81–8.33
3 and more	2.01	1.63–2.47	14.00	11.29–17.35
Adverse obstetric outcomes in previous pregnancy^b^				
Neonatal death				
No	1		1	
Yes	0.61	0.44–0.85	0.44	0.34–0.57
Preterm birth (GA < 37 weeks)				
No	1		1	
Yes	1.16	0.91–1.48	1.40	1.16–1.67
Low birth weight (BW < 2500 g)				
No	1		1	
Yes	0.92	0.74–1.14	1.14	0.97–1.35
Maternal complications^c^				
No	1		1	
Yes	0.99	0.84–1.17	1.19	1.02–1.40

*GA* gestational age, *BW* birth weigh

(^a^) Reference category: intended pregnancy

^b^Excluding primiparous women

^c^Complications in previous pregnancy (eclampsia, high blood pressure, uterine rupture or diabetes)

Similar results were found for unwanted pregnancy, and the strength of the association was generally stronger. Women with an unwanted pregnancy were 4.86 times more likely to report not having a partner. The same effect was observed in relation to parity. Compared with primiparous women, those with up to two prior births were 6.96 times more likely to have an unwanted pregnancy, and those with three or more births were 14.00 times more likely to have an unwanted pregnancy.

Adverse outcomes in previous pregnancies were risk factors for a current unwanted pregnancy. Compared with women who planned the pregnancy at the time, those with previous histories of complication during pregnancy and preterm birth had 40 % and 19 % greater risk, respectively, of unwanted pregnancy. Neonatal death, in contrast, was a protective factor for both mistimed and unwanted pregnancy (OR = 0.61, 95 % CI: 0.44–0.85 and OR = 0.44, 95 % CI: 0.34–0.57, respectively).

## Discussion

The results of this study suggest that Brazil has a high prevalence of unintended pregnancy, which affects more than 50 % of all pregnancies carried to parturition. Similar prevalence is reported in national and international literature, indicating that less than half of women report having the intention to become pregnant at that time [2, 6]. According to estimates based on data from the United Nations Population Division for 2012, approximately 40 % of pregnancies worldwide, or 85 million pregnancies, were unintended. The region with the highest percentage of pregnancies that were unintended was the Latin America and the Caribbean region (56 %), whereas the lowest percentage unintended were found in Africa (35 %) [4]. As mentioned in the introduction, the last nationwide study of pregnancy intendedness in Brazil was carried out in 2006 and showed that 53.9 % of women who had a child in the last 5 years did not plan the pregnancy [14]. More recent research among postpartum women living in a southern city of the country found that 65 % did not plan their pregnancies [18].

In terms of associated factors, unintended pregnancy in this sample was predominantly more likely with younger age, brown and yellow skin color, lower schooling level, not having a partner, not having a paid job, alcohol abuse and tobacco use. The effect of sociodemographic and individual variables is consistent with literature on intention of pregnancy at conception and showed the high degree of social vulnerability of these women [19, 20]. Prietsch and colleagues [18], investigating the factors associated with unplanned pregnancy in a sample of women in Southern Brazil, found statistically significant association between having an unplanned pregnancy and having black and brown skin color, being a teenager, being single, having a low income, being a smoker and having multiparity.

Worldwide, women of childbearing age, regardless of skin color and socioeconomic status, are at risk for experiencing unintended pregnancies, but the extent of that risk is widely variable, with disadvantaged groups of women being the most affected. The health disparities seen in unintended pregnancies must be a main target for social and health policies to reduce inequalities in health [21, 22].

Unintended pregnancy has been linked to many factors, including high risk of unhealthy behaviors. If a woman has an unintended pregnancy, she may be unprepared for it and thus may be less aware of changing her habits, such as improving nutrition or quitting smoking, compared with women with intended pregnancies. These factors may result in less favorable outcomes [1, 23].

Despite being consistent with others studies, the protective effect for unplanned pregnancy found among women aged 35 years and over may reflect recent changes in the fertility behaviour of the Brazilian population. Data from the Brazilian Ministry of Health show that, in the last few years, fertility has been delayed. From 2000 to 2010, the fertility rate among women aged 15 − 19 and 20 − 24 years decreased from 18.8 % to 17.7 % and from 29.3 % to 27.0 %, respectively. Although the latter group still account for the largest portion of fertility in Brazil, the age distribution of fertility was more dispersed in 2010 than in 2000, with increased participation of those over 30 years old [24].

In addition to all of these factors, previous maternal complications and preterm birth revealed strong and significant associations with unintended pregnancy, even after adjusting for sociodemographic and individual risk factors. This relationship with birth outcome was also found by a prospective observational study of 400 postpartum women enrolled at a university medical centre in the United States. In this study, women with a history of prior preterm birth also had a higher chance of unintended pregnancy [12]. A possible explanation is that adverse birth outcomes can trigger anxiety, depression and posttraumatic stress syndrome, all of which are associated with unintended pregnancy [25, 26]. Insufficient contraceptive use might also be related to the risk of unintended pregnancy among depressed women, as those with elevated depression and stress were more likely to be at risk for inconsistent contraceptive use [27]. Another possible explanation is that prematurity is a major risk factor for infant mortality and health complications in newborns, and mothers with previous preterm birth may not feel confident in facing a new pregnancy.

The protective effect of neonatal death related to unwanted and mistimed pregnancy may be explained by different mechanism. Many researchers agree that the intention and decision to have another pregnancy after an adverse outcome such as neonatal death is rife with ambivalent feelings, although 50 %–60 % of mothers wish to become pregnant immediately after this loss [28–30]. Some authors call this decision ‘replacement child syndrome’, characterized by a subsequent pregnancy and birth to substitute for a previous child who has died [31]. Furthermore, there is the belief that a new pregnancy would allow bereaved parents overcome the previous death [29, 32].

In this study, adverse experience in the previous pregnancy was a risk factor for unwanted pregnancy. It is important to understand, beyond the medical approach, the psychological aspects involved in the desire for a new pregnancy and to recognize the contextual factors that impede or facilitate women in their ability to change their behaviour and to choose the moment to be pregnant, as well as the best method to prevent pregnancy.

Family planning in Brazil is an aspect of primary care through the Family Health Strategy [33], but shortcomings remain in terms of access and quality of care. The contraceptive needs of part of the population, particularly women with lower socioeconomic status and education levels and ethnic minorities, are not met, contributing to high rates of unplanned pregnancy in these groups [34]. The low coverage and quality of family planning is caused by several factors. Most professionals working in family planning cite overhead tasks, numbers of appointments incompatible with the available human resources, lack of support material and appropriate physical space for consultations, insufficient training of professionals and outdated information [35]. Conversely, women have often referred to shortages or inadequate information about suitable and available contraceptive methods [36].

The implementation of periodic counselling and guarantee of access to contraceptive methods for all women, especially those at risk for unintended pregnancy, must be a target of an effective family planning policy. Additionally, it is worthwhile to understand the social and cultural context in which unintended pregnancy occurs to avoid to focusing only on individual factors. It is important to integrate social determinants into the causal framework and to create interventions that meet the needs of women and families, especially the most vulnerable. Understanding the persistence of high rates of unplanned pregnancy worldwide, despite the availability of various contraceptive methods, is an important issue for future researches [37].

However before drawing conclusions it is important to consider the strengths and limitations of the current study. The strengths of this study are that it is based on a large, nationwide sample of women and is representative of all births in Brazil in the year it was conducted. It can thus provide reliable and comparable information about unintended pregnancy prevalence and risk factors in Brazil.

There are some limitations. First, the question on pregnancy intention was measured shortly after birth, whereas unwanted pregnancies are more likely to be terminated earlier with induced abortion. Therefore, the prevalence of unintended pregnancy may be underestimated. Second, womens’ intention to become pregnant is a complex process, and knowledge of pregnancy evokes many emotions ranging from worry and fear to happiness and excitement. These emotions may vary during pregnancy and change following the birth, which may have had an impact on women’s responses. Third, in this study the women were not asked about the use of contraceptive methods. We believe this information is important for a better understanding of the factors associated with unplanned pregnancy. However the lack of this variable does not invalidate the other findings of the study. We suggest that future studies examine this relationship.

## Conclusions

The present study has shown that unintended pregnancy is reported by 55.4 % of postpartum women. This study also confirms the findings of previous research on the influence of socioeconomic and individual risk factors on unplanned pregnancy affecting more vulnerable women. Unintended pregnancy may influence different women in different ways, according to their income, race/skin color, marital status and other characteristics. Moreover, the results of this study include a new approach to the problem, revealing the importance of negative outcomes in previous pregnancies as risk factors for unplanned pregnancy. In view of the high prevalence of unintended pregnancy in national studies (and confirmed by this research), it is necessary to overcome barriers and increase the access and quality of family planning services, including the psychosocial approach, to ensure greater adherence to available contraceptive methods.

### Portuguese version

A Portuguese translation of this article is available as Additional file 1.

### Peer review

The reviewer reports for this article are available as Additional file 2.

## Additional files

Additional file 1: Translated article. (PDF 697 kb)
Additional file 2: Reviewer report. (PDF 168 kb)
