# Supplementary material for: Factors associated with unintended pregnancy in Brazil: cross-sectional results from the Birth in Brazil National Survey, 2011/2012
Source: Reprod Health. 2016 Oct 17;13(Suppl 3):118. doi: 10.1186/s12978-016-0227-8 (PMC5073899; doi:10.1186/s12978-016-0227-8)
Supplement: Additional file 1: — Translated article. (PDF 697 kb) [file 12978_2016_227_MOESM1_ESM.pdf]

Fatores associados à gravidez não planejada no Brasil: resultados da Pesquisa Nascer no Brasil, 2011/2012.

Resumo:

Introdução: A gravidez não planejada é um grave problema de saúde pública no Brasil. Ela é referida por mais da metade das mulheres que tiveram filhos, mas as características dessas mulheres é pouco conhecida. O objetivo deste estudo foi analisar a prevalência e a associação entre o planejamento da gestação com um conjunto de variáveis sociodemográficas, individuais e de antecedentes obstétricos.

Método: A pesquisa Nascer no Brasil é um estudo seccional com representatividade nacional, que entrevistou 23.894 puérperas. A informação sobre o planejamento da gravidez foi obtida na maternidade após o parto, sendo classificada em três categorias: queria engravidar, queria esperar mais tempo e não queria engravidar. Inicialmente foi testada a associação entre variáveis sociodemográficas, características individuais e antecedentes obstétricos com o planejamento da gestação utilizando-se modelo de regressão multinomial simples, calculando-se a razão de chances. As variáveis significativas na análise bivariada foram incluídas no modelo de regressão multinomial multivariado, mantendo-se no modelo final aquelas que apresentaram significância estatística menor que 5%.

Resultados: A gravidez não planejada foi referida por 55,4% das puérperas. Associou-se com querer esperar mais tempo para engravidar ter menos de 20 anos (OR = 1,89, IC 95%: 1,68–2,14); cor da pele parda (OR = 1,15, IC 95%: 1,04–1,27) ou amarela (OR = 1,56, IC 95%: 1,05–2,32); não ter companheiro (OR = 2,32, IC 95%: 1,99–2,71); não ter trabalho remunerado (OR = 1,15, IC 95%: 1,04–1,27); uso abusivo de bebidas alcoólicas (OR = 1,25, IC 95%: 1,04–1,50) e ter três ou mais filhos (OR = 2,01, IC 95%: 1,63–2,47). Os mesmos fatores associaram-se com não querer engravidar, e a

força de associação foi geralmente mais forte. Mulheres com três ou mais partos anteriores apresentaram 14 vezes a chance de não querer engravidar e aquelas com complicações em gestações anteriores ou história de parto prematuro apresentaram chance 40% e 19% maior, respectivamente. Antecedente de óbito neonatal foi um fator de proteção tanto para aquelas que queriam esperar mais tempo (OR = 0,61, IC 95%: 0,44–0,85) quanto para as que não queriam engravidar (OR = 0,44, IC 95%: 0,34–0,57). **Conclusão:** O estudo confirmou a importância dos fatores de risco socioeconômicos e individuais no planejamento da gravidez, relatado também em outras pesquisas. Além disso, trouxe um conhecimento novo sobre a importância dos antecedentes obstétricos como óbito neonatal, prematuridade e complicações em gestações anteriores como fatores de risco para uma gravidez não planejada.

Palavras-chave: gravidez não planejada; fatores de risco; epidemiologia; Brasil.

Keywords: unintended pregnancy; unplanned pregnancy; risk factors; epidemiology; Brazil

Introdução:

Por uma série de razões sociais e econômicas, a maioria dos casais deseja planejar o momento e o espaçamento da gravidez, evitando uma gestação indesejada. Entretanto, a gravidez não planejada para aquele momento (queria esperar mais tempo), ou não desejada em nenhum momento (não queria engravidar) [1], é um problema de saúde nos países desenvolvidos e de média e baixa renda [2,3]. Em 2012, a taxa de gravidez não planejada no mundo foi de 53 por 1000 mulheres de 15 a 44 anos de idade. A taxa mais elevada foi observada na África (80 por 1000), e a menor na Europa e Oceania (43 por 1000) [4]. Nos Estados Unidos, quase metade das gestações não são

planejadas [5].

Vários fatores de risco são identificados com o não planejamento da gravidez. A literatura aponta como principais fatores a baixa condição socioeconômica, falta de apoio social, idade materna, parição e hábitos maternos como tabagismo e consumo de bebidas alcoólicas [6,7]. Esses fatores têm se mostrado estáveis nas diferentes culturas. Uma consequência frequente da gravidez não planejada é a sua interrupção por meio do aborto, muitas vezes realizado em condições não seguras, particularmente nos países em que esta prática é considerada ilegal. Além disso, a gravidez não planejada está associada com desfechos maternos e neonatais desfavoráveis como o início tardio do pré-natal e parto prematuro, além de efeitos negativos sobre a saúde física e mental da mãe e da criança [8-10]. Todos estes fatores aumentam os custos do cuidado neonatal e estão associados com incapacidades de longo prazo para as mulheres e os bebês [11,12].

No Brasil, a gravidez não planejada permanence elevada a despeito da drástica redução da taxa de fecundidade observada nas últimas décadas, atingindo 1,8 filhos por mulher em idade fértil em 2011[13], e do uso disseminado de métodos contraceptivos. Segundo o última Pesquisa Nacional de Demografia e Saúde (PNDS), realizada em 2006, 67,8% das mulheres que tiveram relações sexuais nos últimos 12 meses estavam usando algum método contraceptivo moderno. A pesquisa também revelou que 53,9% de todos os nascimentos ocorridos nos últimos 5 anos não foram planejados [14]. No Brasil, o aborto é uma prática legal somente em casos de estupro, quando há risco de vida para a mãe, ou o feto apresenta anencefalia ou outra anomalia congênita incompatível com a vida extra-uterina. Consequentemente, a taxa de nascimentos decorrentes de gestações não planejadas é elevada [11].

Considerando a importância da gravidez não planejada sobre a saúde da mulher e seus filhos, o objetivo deste estudo foi analisar a associação entre o planejamento da

gravidez com variáveis sociodemográficas, fatores individuais e história obstétrica entre as mulheres que tiveram seus bebês nos hospitais incluídos na pesquisa Nascer no Brasil, entre 2011 e 2012.

Métodos

Amostra e desenho do estudo

A pesquisa Nascer no Brasil é um estudo com representatividade nacional, que entrevistou 23.894 mulheres após o parto e coletou informações dos prontuários das mães e seus filhos, no período de fevereiro de 2011 a outubro de 2012. A amostra foi selecionada em três estágios. Primeiro foram selecionados os hospitais que apresentaram 500 ou mais nascimentos em 2007. Os hospitais foram classificados de acordo com as cinco macrorregiões (norte, nordeste, sudeste, sul e centro-oeste), localização (capital e não-capital) e tipo de hospital (público, misto ou privado). No segundo estágio foi calculado o número de dias necessários para alcançar o tamanho fixo de 90 puérperas em cada hospital selecionado (mínimo de sete dias em cada hospital). No último estágio foram selecionadas 90 puérperas em cada hospital da amostra. A amostra foi distribuída em 266 hospitais em 191 municípios, incluindo todas as capitais. Consideramos elegíveis todas as puérperas que deram à luz a um nascido vivo, independente do peso e idade gestacional, ou nascido morto com peso superior a 500 gramas ou idade gestacional maior que 22 semanas.

Na primeira fase do estudo foram realizadas entrevistas face a face durante a internação para o parto, coletadas informações do prontuário das mães e seus filhos, e fotografados os cartões de pré-natal. Formulários eletrônicos foram desenvolvidos e

validados para a coleta de dados, além de manuais com a descrição dos procedimentos da pesquisa, a fim de garantir a qualidade dos dados e minimizar os erros sistemáticos. As entrevistas foram realizadas por entrevistadores treinados pela coordenação da pesquisa e os supervisores do trabalho de campo replicaram 5% da amostra.

Por se tratar de uma amostra complexa, utilizou-se procedimentos de calibração e pesos amostrais para garantir a coerência das estimativas amostrais com os totais populacionais obtidos de fonte externa. Maiores detalhes sobre a amostra podem ser encontrados em Vasconcellos et al. [15].

Variáveis do estudo

A variável desfecho foi o planejamento da gestação. As mulheres foram questionadas se a gravidez foi planejada, com três possibilidades de resposta: queria engravidar, queria esperar mais tempo ou não queria engravidar.

As seguintes variáveis foram incluídas no estudo para testar a associação com gravidez não planejada:

- Variáveis sociodemográficas: idade da mãe no momento do parto (< 20; 20–34; ≥ 35); cor da pele (branca, preta, parda, amarela); e anos de estudo (≤7; 8–11; ≥ 12). As categorias de cor da pele basearam-se na classificação do Censo Demográfico.
- Variáveis individuais: situação conjugal (com ou sem companheiro); ter trabalho remunerado; consumo de bebidas alcoólicas e tabagismo; e parição (primípara, um filho, dois filhos, e três filhos ou mais)
- Desfechos obstétricos em gestações anteriores: óbito neonatal; natimorto; baixo peso (peso ao nascer < 2.500g), prematuridade (idade gestacional

< 37 semanas) e complicações graves durante a gestação (eclampsia, diabetes, hipertensão arterial e rutura uterina).

O consumo de bebidas alcoólicas foi aferido utilizando-se o instrumento TWEAK, originalmente desenvolvido para identificar o hábito de ingestão de bebidas alcoólicas em gestantes [16]. Mulheres que obtiveram escore maior ou igual a três foram consideradas com risco de alcoolismo. A variável foi classificada em três categorias: não ingeriu bebidas alcoólicas na gestação; consumiu bebidas alcoólicas mas sem apresentar risco de alcoolismo; e consumiu bebidas alcoólicas com risco de alcoolismo. Foram consideradas fumantes as mulheres que relataram fumar pelo menos um cigarro por dia durante toda a gestação.

Análise dos dados

Inicialmente descrevemos as características das mulheres segundo o planejamento da gestação (queria engravidar, queria esperar mais tempo, não queria engravidar). Para estimar a associação com as variáveis do estudo, utilizamos modelo de análise multinomial, calculando-se as razões de chances e seus intervalos de confiança de 95%, tendo como categoria de referência "queria engravidar". Todas as variáveis significativas na análise bivariada foram incluídas no modelo multinomial multivariado utilizando-se análise hierarquizada. Em cada bloco de variáveis (sociodemográficas, individuais e antecedents obstétricos), as variáveis significativas foram utilizadas para ajuste das variáveis do mesmo bloco e do bloco seguinte, retendo-se no modelo final as variáveis que permaneceram significativas ao nível de 5%.

As análise foram realizadas com o pacote estatístico SPSS 17.0 (Chicago, Il, USA), e por ser tratar de uma amostra complexa utilizamos o módulo *Complex Sample* para correção do efeito de desenho. O estudo seguiu as recomendações do STROBE

(Strengthening the Reporting of Observational Studies in Epidemiology) para análise de estudos seccionais [17].

Considerações éticas

As entrevistas hospitalares foram realizadas após a assinatura do Termo de Consentimento Livre e Esclarecido (TCLE) que incluía a autorização para contatos telefônicos posteriores. A pesquisa Nascer no Brasil foi aprovada pelo Comitê de Ética em Pesquisa, da Escola Nacional de Saúde Pública Sérgio Arouca, sob o número CAAE 0096.0.031.000-10.

Resultados

Foram entrevistadas 23.894 puérperas. A prevalência de gravidez não planejada no estudo foi de 55,4%. A proporção de mulheres que referiu querer esperar mais tempo ou não queria engravidar foi 25,5% e 29,9%, respectivamente .

A Tabela 1 apresenta as características sociodemográficas, individuais e obstétricas do total da amostra e para os três grupos analisados (queria engravidar, queria esperar mais tempo, não queria engravidar) A maioria das mulheres tinha entre 20 a 34 anos de idade (70,4%), era de cor parda (54,9%), tinha 12 anos ou mais de estudo (39%), vivia com companheiro (81,4%), não tinha trabalho remunerado (59,7%) e era primípara (4,5%). Em relação aos hábitos não saudáveis, 10% referiram consumo de bebidas alcoólicas e 9,6% consumo de cigarros durante a gestação.

Em relação à história obstétrica (excluindo a gestação atual e somente entre as multíparas), os eventos mais frequentes foram a prematuridade (11,9%), baixo peso ao nascer (13,0%) e complicações graves na gestação (13,7%). Perda fetal e óbito neonatal foi referida por 4,4% e 3,4% das mulheres, respectivamente. É importante enfatizar que

estes resultados referem-se à gestações que terminaram no parto e todas as mulheres foram entrevistadas após o parto. Interrrupções da gestação por aborto não foram incluídas no estudo.

Um perfil bastante distinto foi observado entre as mulheres que queriam engravidar em relação àquelas que queriam esperar mais tempo ou não queriam engravidar. Comparando-se os três grupos, as mulheres que queriam engravidar apresentavam maior proporção de idade ≥ 35 years (52%), eram majoritariamente de cor da pele branca (52,7%), elevado nível de escolaridade (59,3% com 12 anos ou mais de estudo), tinham companheiro (49,5%) e eram primíparas (51,5%). Entretanto, elevada proporção de hábitos não saudáveis, como uso de bebidas alcoólicas e fumo, foi observada em todos os grupos, mostrando forte associação destes hábitos entre as mulheres que não queriam engravidar. Em relação aos desfechos adversos em gestações anteriores entre as multíparas, aquelas com história de natimorto ou neomorto apresentaram maior chance de querer engravidar. Mulheres com relato de nascimento de filhos prematuros ou com baixo peso, e aquelas que apresentaram complicações em gestações anteriores, apresentaram maior chance de não querer engravidar.

A Tabela 2 apresenta os resultados da análise de regressão multinomial bivariada. Todas as categorais das variáveis sociodemográficas e individuais mostraram associação com a gravidez não planejada e foram incluídas no modelo multinomial multivariado. Querer esperar mais tempo ou não querer engravidar apresentou associação inversa e significativa com óbito neonatal prévio (OR = 0,61, IC 95%: 0,45–0,81 and OR = 0,61, IC 95% : 0,48–0,76, respectivamente). Não querer engravidar mostrou associação positiva e significante com história de baixo peso (OR = 1,37, IC

95%: 1,18–1,59), prematuridade (OR = 1,44, IC 95%: 1,23–1,69) e complicações em gestações anteriores (OR = 1,21, IC 95%: 1,04–1,41).

A análise multivariada ajustada para cada bloco de variáveis (sociodemográfica, individual e obstétrica) confirmou os resultados da análise bivariada. Todas as variáveis que mantiveram associação com pelo menos uma categoria sob análise entre as mulheres que queriam esperar mais tempo ou não queriam engravidar entraram no modelo multivariado final, para identificar os fatores de risco da gravidez não planejada no Brasil.

O modelo final é apresentado na Tabela 3. As seguintes variáveis mantiveram associação positiva e estatisticamente significativa com querer esperar mais tempo: idade materna menor que 20 anos (OR = 1,89, IC 95%: 1,68–2,14); cor da pele parda (OR = 1,15, IC 95%: 1,04–1,27) ou amarela (OR = 1,56, IC 95%: 1,05–2,32); não ter companheiro (OR = 2,32, IC 95%: 1,99–2,71); não ter trabalho remunerado (OR = 1,15, IC 95%: 1,04–1,27); risco de alcoolismo (OR = 1,25, IC 95%: 1,04–1,50) e parição. Quanto maior o número de filhos, maior a chance de querer esperar mais tempo.

Resultados semelhantes foram observados entre as mulheres que não queriam engravidar, e a força da associação foi geralmente mais forte. Mulheres sem companheiro apresentaram 4,86 vezes a chance de não querer engravidar em relação àquelas que tinham companheiro. O mesmo efeito foi observado em relação à parição. Em comparação às primíparas, mulheres com dois filhos ou três filhos ou mais apresentaram, respectivamente, 6,96 e 14 vezes a chance de não querer engravidar.

Desfechos adversos em gestações anteriores foram fatores de risco para uma gestação não desejada. Comparada com as mulheres que queriam engravidar, aquelas com história prévia de complicações na gestação e parto prematuro apresetaram 40% e

19% mais chance, respectivamente, de não querer engravidar. Óbito neonatal, por outro lado, foi um fator de proteção tanto para querer esperar mais tempo como não querer engravidar (OR = 0,61, IC 95%: 0,44–0,85 e OR = 0,44, IC 95%: 0,34–0,57, respectivamente).

Discussão

Os resultados deste estudo sugerem que o Brasil tem uma alta prevalência de gravidez não planejada, que afeta mais de 50% das gestações que chegam ao parto [2,6]. Resultados semelhantes são descritos na literatura nacional e internacional, indicando que menos da metade das mulheres relatam intenção de engravidar no momento em que ficam grávidas. De acordo com estimativas das Nações Unidas para 2012, cerca de 40% das gestações no mundo, ou 85 milhões de gestações, não foram planejadas. As regiões com maiores prevalências de gravidez não planejada foram a América Latina e o Caribe (56%), enquanto a mais baixa foi encontrada na África (35%) [4]. Como mencionado na introdução, o último estudo sobre planejamento reprodutivo no Brasil foi realizado em 2006 e mostrou que 53,9% das mulheres que tiveram filhos nos últimos cinco anos não planejaram a gravidez [14]. Mais recentemente, pesquisa com puérperas residentes em uma cidade da região sudeste do país mostrou que 65% não planejaram a gravidez [18].

Em relação aos fatores associados, a gravidez não planejada nesta amostra foi mais frequente entre as mulheres jovens, de cor parda ou amarela, de baixa escolaridade, sem companheiro, sem trabalho remunerado, fumantes e que fazem uso abusivo de bebidas alcoólicas. O efeito das variáveis sociodemográficas e individuais foi consistente com a literatura sobre o tema e mostrou o grau de vulnerabilidade social

destas mulheres [19-20]. Prietsch e colaboradores [18], investigando os fatores associados com a gravidez não planejada em uma amostra no sudeste do Brasil encontrou associação estatisticamente significativa entre as mulheres com a cor da pele preta ou parda, adolescente, solteira, baixa renda, fumante e multípara.

No mundo, mulheres em idade fértil, independente da cor da pele e da situação socioeconômica encontram-se em risco de ter uma gravidez não planejada, porém a magnitude deste risco é variável, afetando principalmente o grupo de mulheres em maior desvantagem social [21,22].

A gravidez não planejada tem sido associada a vários fatores, incluindo hábitos não saudáveis. A mulher que vivencia uma gestação não planejada pode estar menos preparada para assumir mudanças de comportamento como adotar alimentação mais saudável ou parar de fumar quando comparada com aquelas com gravidez planejada. Estes fatores podem resultar um desfechos menos favoráveis [1,23].

A despeito da consistência com outros estudos, o efeito protetor da idade acima de 35 anos pode refletir as recentes mudanças no comportamento da fecundidade na população brasileira. Dados do Ministério da Saúde mostram que a taxa de fecundidade vem reduzindo nos últimos anos. De 2000 a 2010, a taxa de fecundidade entre as mulheres de 15 a 19 anos caiu de 18,8% para 17,7%, e entre as mulheres de 20 a 24 anos passou de 29,3% para 27%. Embora o grupo etário de 20 a 24 anos contribua com a maior parcela da fecundidade no Brasil, a distribuição da fecundidade mostrava-se mais espalhada em 2010 do que em 2000, com aumento da participação das mulheres com 30 anos ou mais [24].

Além desses fatores, complicações em gestações anteriores e prematuridade apresentaram associação forte e significativa com a gravidez não planejada, mesmo

após o ajuste para as variáveis sociodemográficas e individuais. Esta associação também foi observada em estudo observacional prospectivo com 400 puérperas em um centro médico universitário nos Estados Unidos. Nesse estudo, mulheres com história prévia de parto prematuro também apresentaram maior chance de gravidez não planejada [12]. Uma possível explicação é que os resultados adversos de uma gestação possam agir como gatilho para ansiedade, depressão e síndrome do estresse pós-traumático, eventos associados com o planejamento reprodutivo [25,26]. Mulheres com depressão e estresse apresentam maior probabilidade de uso inadequado de métodos contraceptivos, aumentando o risco de gravidez não planejada [27]. Outra possível explicação é que a prematuridade é o principal fator de risco para a mortalidade infantil e complicações no recém-nascido, e mães que passaram por esta experiência podem não se sentir seguras frente à uma nova gestação.

O efeito protetor da mortalidade neonatal para a gravidez não planejada pode ser explicada por diferentes mecanismos. Muitos pesquisadores acreditam que a intenção e decisão de ter uma gestação após uma experiência negativa como óbito neonatal é acompanhada de sentimentos de ambivalência, embora 50% a 60% das mães desejem nova gravidez após a perda de um bebê [28-30]. Alguns autores denominam esta decisão de “síndrome do bebê de substituição”, caracterizada por uma gravidez que vem substituir uma perda anterior [31]. Acredita-se que uma nova gravidez permitiria aos pais superar o luto pela morte de um filho [29,32].

Neste estudo, a experiência adversa em gestação anterior foi um fator de risco para uma gravidez não planejada. É importante compreender, além da abordagem médica, os aspectos psicológicos envolvidos no desejo de uma nova gestação e reconhecer os fatores contextuais que impedem ou facilitam a

capacidade da mulher de mudar seu comportamento e escolher o melhor momento para engravidar, assim como o melhor método para evitá-la.

O planejamento familiar é uma atribuição da atenção primária em saúde no Brasil por meio da Estratégia de Saúde da Família [33], entretanto ainda permanecem problemas de acesso e qualidade do cuidado. As necessidades de contracepção por parte da população, particularmente das mulheres em pior situação socioeconômica, baixa escolaridade e minorias étnicas, ainda não são adequadamente atendidas, contribuindo para elevada proporção de gravidez não planejada nesses grupos [34]. Vários fatores podem explicar a baixa cobertura e qualidade do planejamento familiar. Muitos profissionais que trabalham nesta área citam a sobrecarga de trabalho, número de consultas incompatíveis com a disponibilidade de recursos humanos, falta de suporte material e espaço físico adequado para as consultas, treinamento insuficiente dos profissionais e informações desatualizadas [35]. Por outro lado, as mulheres referem frequentemente pouca informação ou informação inadequada sobre os métodos contraceptivos disponíveis [36].

A implementação do aconselhamento periódico e a garantia de acesso aos métodos contraceptivos por todas as mulheres, especialmente para aquelas em maior risco de ter uma gravidez não planejada, deve ser o foco de uma política de planejamento familiar efetiva. Adicionalmente, deve-se compreender o contexto social e cultural em que a gravidez não planejada ocorre evitando-se atuar somente sobre fatores individuais. É importante integrar os determinantes sociais à rede de causalidade e criar intervenções que preencham as necessidades das mulheres e sua família, especialmente as mais vulneráveis. Compreender a persistência da elevada taxa de gravidez não planejada no mundo, a despeito da disponibilidade de vários métodos

contraceptivos, é uma questão importante para pesquisas futuras [37].

Apesar dos resultados relevantes é importante apontarmos as forças e limitações deste estudo. Sua principal força reside no fato de ser uma investigação baseada em uma amostra grande e representativa de todos os nascimentos no Brasil quando o estudo foi realizado, fornecendo resultados confiáveis e comparáveis sobre a prevalência e fatores de risco associados com o planejamento reprodutivo no Brasil.

Algumas limitações devem ser apontadas. Primeiro, a pergunta sobre a intenção de engravidar foi feita imediatamente após o parto, e gravidezes não planejadas podem ser interrompidas precocemente por abortos. Portanto, a prevalência de gravidez não planejada pode estar subestimada. Segundo, a intenção de engravidar é um processo complexo, e o diagnóstico da gravidez evoca muitas emoções que variam da preocupação e medo, a grande felicidade e emoção. Esses sentimentos variam durante a gravidez e mudam com o nascimento, o que pode ter influenciado as respostas das mulheres. Terceiro, neste estudo não foi questionado o uso de métodos contraceptivos. Acreditamos que esta informação seja importante para melhor compreensão dos fatores associados à gravidez não planejada. Entretanto, a falta desta variável não invalida os achados deste estudo. Sugerimos que pesquisas futuras examinem esta relação.

Conclusões

Este estudo revelou que a gravidez não planejada foi referida por 55,4% das puérperas entrevistadas. Ele também confirmou os achados de estudos prévios sobre a influência dos fatores de risco socioeconômicos e individuais na gravidez não planejada, afetando as mulheres mais vulneráveis. A gravidez não planejada pode afetar mulheres

de formas diferentes, segundo sua renda, cor da pele, situação conjugal e outras características. Além disso, os resultados do estudo incluem uma nova abordagem sobre o problema revelando a importância de desfechos negativos em gestações anteriores como fatores de risco para a gravidez não planejada. Face a elevada prevalência de gravidez não planejada em estudos nacionais (e confirmada nesta pesquisa), é necessário superar as barreiras e aumentar o acesso e a qualidade dos serviços de planejamento familiar, incluindo abordagem psicológica, e garantir maior adesão aos métodos contraceptivos disponíveis.

Conflito de interesse

Os autores negam conflitos de interesse.

Contribuição dos autores

MMTF concebeu o estudo, delineou e realizou as análises, escreveu o artigo e foi responsável por sua versão final. MLB e ACSAF delinearam o estudo, realizaram as análises e colaboraram na redação do artigo. SA colaborou na redação do artigo e revisou sua versão final. SGNG and MCL coordenaram a pesquisa Nascer no Brasil, colaboraram na redação do artigo e revisaram a versão final. Todos os autores colaboraram na interpretação dos resultados e aprovaram a versão final.

Agradecimentos

Os autores agradecem a o apoio financeiro do CNPq, Faperj, DECIT/MS e da Escola Nacional de Saúde Pública da Fundação Oswaldo Cruz, assim como a todos os hospitais que participaram da pesquisa, a equipe de trabalho de campo e às mães que gentilmente concordaram em participar.

Referências

1. Mosher WD, Jones J, Abma JC. Intended and Unintended Births in the United States: 1982–2010. National health statistics reports; no 55. Hyattsville, MD: National Center for Health Statistics. 2012.
2. Exavery A, Kanté AM, Hingora A, Mbaruku G, Pemba S, Phillips JF. How mistimed and unwanted pregnancies affect timing of antenatal care initiation in three districts in Tanzania. BMC Pregnancy Childbirth. 2013, 13:35.
3. Lukasse M, Laanpere M, Karro H, Kristjansdottir H, Schroll AM, Van Parys AS et al. On behalf of the Bidens study group. Pregnancy intendedness and the association with physical, sexual and emotional abuse – a European multi-country cross-sectional study. BMC Pregnancy and Childbirth. 2015, 15:120.
4. Sedgh G, Singh S, Hussain R. Intended and Unintended Pregnancies Worldwide in 2012 and Recent Trends. Stud Fam Plann. 2014;45(3):301-314.
5. Kelly PJ. Unintended Pregnancy and the Social Determinants of Health. Public Health Nurs. 2014;31(5):385–386.
6. Finer LB, Zolna MR. Unintended pregnancy in the United States: Incidence and disparities. Contraception. 2011;84(5):478-485.

7. Langer A. El embarazo no deseado: impacto sobre la salud y la sociedad en América Latina y el Caribe. Pan Am J Public Health. 2002;11(3): 192-205.
8. Gipson JD, Koenig MA, Hindin MJ. The effects of unintended pregnancy on infant, child, and parental health: a review of the literature. Stud Fam Plann. 2008; 39:18-38.
9. Kost K, Lindberg L. Pregnancy intentions, maternal behaviors, and infant health: investigating relationships with new measures and propensity score analysis. Demography. 2015; 52(1):83-111.
10. Faisal-Cury A, Menezes PR, Quayle J, Matijasevich A. Unplanned pregnancy and risk of maternal depression: secondary data analysis from a prospective pregnancy cohort, Psychology, Health & Medicine. 2016 DOI:10.1080/13548506.2016.1153678
11. Le HH, Connolly MP, Bahamondes L, Cecatti JG, Yu J, Hu HX. The burden of unintended pregnancies in Brazil: a social and public health system cost analysis. International Journal of Women's Health. 2014;6:663-670.
12. Shah PS, Balkhair T, Ohlsson A, Beyene J, Scott F, Frick C. Intention to Become Pregnant and Low Birth Weight and Preterm Birth: A Systematic Review. Matern Child Health J. 2011;15:205–216.
13. Departamento de Informática do SUS (Datasus). Indicadores e Dados Básicos (IDB 2012). Brasil; 2012. http://tabnet.datasus.gov.br/cgi/idb2012/a05b.htm accessed 10th Jun, 2015.
14. Brasil. Pesquisa Nacional de Demografia e Saúde da Criança e da Mulher – PNDS 2006: dimensões do processo reprodutivo e da saúde da criança. Ministério da Saúde. Brasília; 2009.

15. Vasconcellos MTL, Silva PLN, Esteves-Pereira AP, Schilithz AOC, Souza Junior PRB, Szwarcwald CL. Sampling design for the Birth in Brazil: National Survey into Labour and Birth. Cad Saúde Pública. 2014;30 Suppl 1:S49-S58.

16. Sarkar M, Einarson G, Koren C. 2010. Comparing of effectiveness of TWEAK and T-ACE in determining problem drinkers in pregnancy. Alcohol & Alcoholism. 2010, 45(4):356-360.

17. Von Elm E, Altman DG, Egger M, Pocock S J, Gøtzsche PC, Vandenbroucke JP, & for the STROBE Initiative. The Strengthening the Reporting of Observational Studies in Epidemiology (STROBE) Statement: Guidelines for Reporting Observational Studies. *PLoS Medicine*. 2007;4(10):e296.

18. Prietsch SOM, González-Chica DA, Cesar JA, Mendoza-Sassi RA. Gravidez não planejada no extremo Sul do Brasil: prevalência e fatores associados. Cad Saúde Pública. 2011; 27(10):1906-1916.

19. Shreffler KM, Greil AL, Mitchell KS, McQuillan J. Variation in pregnancy intendedness across U.S. women's pregnancies. Matern Child Health J. 2015;19(5):932–8.

20. Powers JR, Mc Dermott LJ, Loxton DJ, Chojenta CL. A Prospective Study of Prevalence and Predictors of Concurrent Alcohol and Tobacco Use During Pregnancy Matern Child Health J. 2013; 17:76–84.

21. Ikamari L, Izugbara C, Ochako R. Prevalence and determinants of unintended pregnancy among women in Nairobi, Kenya BMC Pregnancy Childbirth. 2013, 13:69.

22. Levi A & Dau KQ. Meeting the National Health Goal to Reduce Unintended Pregnancy. J Obstet Gynecol Neonatal Nurs. 2011;40: 775-781.

23. Cheng D, Schwarz EB, Douglas E, Horon I. Unintended pregnancy and associated maternal preconception, prenatal and postpartum behaviors. Contraception. 2009;79:194–8.

24. Brasil. Health Brazil 2011: analysis of the health situation and the surveillance of the woman's health. Ministério da Saúde. Brasília, 2012.

25. Grekin R, O'Hara MW. Prevalence and risk factors of postpartum post traumatic stress disorder: a meta-analysis. Clin Psychol Rev. 2014;34(5):389-401.

26. Patel M, Bailey RK, Jabeen S, Ali S, Barker N, Osiezagha K. Postpaartum Depression: A Review. Journal of Heath Care for the Poor and Underserved. 2012;23(1):534-542.

27. Stidham Hall K, Moreau C, Trussell J, Barber J. Young women's consistency of contraceptive use–does depression or stress matter? Contraception. 2013;88(5):641–9.

28. Mills T, Ricklesford C, Cooke A, Heazell A, Whitworth M, Lavender T. Parents' experiences and expectations of care in pregnancy after stillbirth or neonatal death: a metasynthesis. Int J Obstet Gynaecol. 2014;121(8):943–50.

29. Osis MJD, Faúndes A, Makuch MY, Mello MB, Sousa MH, Araujo MJO. Family planning in Brazil today: an analysis of recent research. Cad Saúde Pública. 2006;22(11):2481-2490.

30. Estok P, Lehman A. Perinatal Death: Grief Support for Families. Birth. 1983;10(1):17–28.

31. Robertson PA, Kavanaugh K. Supporting parents during and after a pregnancy subsequent to a perinatal loss. J Perinat Neonatal Nurs. 1998;12(2):63–71.

32. Frøen JF, Cacciatore J, McClure EM, Kuti O, Jokhio AH, Islam M, et al.

Stillbirths: why they matter. Lancet Lond Engl. 2011;377(9774):1353–66.

33. Brasil. Saúde sexual e saúde reprodutiva. Ministério da Saúde. Brasília, 2013.

34. Tavares LS, Leite IC, Telles FSP. Necessidade insatisfeita por métodos anticoncepcionais no Brasil. Rev Bras Epidemiol. 2007;10(2):139–48.

35. Moura ERF, Sousa RA. Education in reproductive health: proposal or reality in the Family Health Program? Cad Saúde Pública. 2002;18(6):1809–11.

36. Santos JC, Freitas PM. Family planning under the view of development. Ciênc Saúde Coletiva. 2011;16(3):1813–20.

37. Kavanaugh, ML. Moving beyond the individual in reproductive health: Exploring the social determinants of unintended pregnancy. PhD thesis. University of Pittsburgh, Department of Behavioral and Community Health. 2008. http://d-scholarship.pitt.edu/8006/1/KavanaughML_etd2008.pdf accessed 15th Jun, 2015.

Tabela 1 – Planejamento da gravidez segundo variáveis sociodemográficas, individuais e antecedentes obstétricos. Nascer no Brasil, 2011/2012.

Variáveis	Total		Queria engravidar	Queria esperar mais	Não queria engravidar
	N	%	%	%	%
**Variáveis sociodemográficas**					
Idade (anos)					
< 20	4571	19,1	34,7	33,5	31,8
20 - 34	16807	70,4	46,2	25,3	28,6
≥ 35	2509	10,5	52,0	12,8	35,2
Anos de estudo					
≤ 7 anos	6017	26,6	39,6	21,4	39,1
8 - 11 anos	12537	25,6	44,6	26,5	28,9
≥12 anos	5472	39,0	59,3	24,8	16,0
Cor da pele					
Branca	8701	36,2	52,7	23,8	23,5
Preta	1879	7,8	40,8	23,1	36,1
Parda	13191	54,9	43,7	25,6	30,7
Amarela	265	1,1	41,3	29,9	28,8
**Variáveis individuais**					
Situação conjugal					
Com companheiro	19440	81,4	49,5	25,0	25,5
Sem companheiro	4432	18,6	22,9	28,0	49,1
Trabalho remunerado					
Não	14272	59,7	40,5	26,6	32,8
Sim	9616	40,3	50,5	23,9	25,6
Consumo de bebidas alcoólicas					
Não	20070	86,0	46,2	25,5	28,3
Sim, mas sem risco de alcoolismo	922	4,0	43,1	24,8	32,2
Sim, mas com risco de alcoolismo	2340	10,0	34,3	26,4	39,3
Tabagismo					
Não	21579	90,4	45,7	26,2	28,1
Sim	2296	9,6	33,7	19,5	46,8
Parição					
Primípara	9910	41,5	51,5	29,4	19,1
1 filho	6697	28,0	46,6	26,0	27,5
2 filhos	3702	15,5	33,0	19,1	47,9
3 filhos e mais	3575	15,0	22,5	14,9	62,6
**Desfechos obstétricos adversos em gestações anteriores**[a]					
Óbito nenonatal					
Não	12254	96,6	38,1	22,2	39,7
Sim	432	3,4	50,3	17,8	31,9

Natimorto					
Não	12129	95,6	35,7	16,6	32,7
Sim	558	4,4	41,3	20,5	38,3
Prematuridade (IG < 37 semanas)					
Não	11176	88,1	39,2	22,4	38,4
Sim	1510	11,9	33,2	19,9	46,9
Baixo peso ao nascer (< 2500g)					
Não	11039	87,0	39,1	22,6	38,4
Sim	1648	13,0	34,5	19,0	46,5
Complicações maternas[b]					
Não	10774	86,3	38,7	22,5	38,8
Sim	1717	13,7	36,2	19,8	44,0

IG = idade gestacional

[a]Excluídas as primíparas

[b]Complicacões em gestações anteriores (eclampsia, hipertensão arterial, rutura uterina ou diabetes)

Tabela 2 – Associação bruta entre as variáveis do estudo e o planejamento da gestação. Nascer no Brasil, 2011/2012.

**Variável**	**Queria esperar mais([a])**		**Não queria engravidar([a])**	
	**OR**	**IC 95%**	**OR**	**IC 95%**
**Variáveis sociodemográficas**				
Idade (anos)				
< 20	1,76	1,57–1,98	1,48	1,32–1,67
20 - 34	0,45	0,39–0,52	1,09	0,97–1,24
≥ 35	1	-	1	-
Anos de estudo				
≤ 7 anos	1,21	1,05–1,39	3,37	2,94–3,88
8 - 11 anos	1,32	1,16–1,50	2,29	2,01–2,62
≥12 anos	1		1	
Cor da pele				
Branca	1	-	1	-
Preta	1,19	1,00–1,42	1,75	1,49–2,06
Parda	1,26	1,15–1,40	1,49	1,35–1,65
Amarela	1,66	1,14–2,42	1,67	1,18–2,32
**Variáveis individuais**				
Situação conjugal				
Com companheiro	1	-	1	-
Sem companheiro	2,42	2,04–2,87	4,17	3,59–4,85
Trabalho remunerado				
Não	1	-	1	-
Sim	1,39	1,26–1,53	1,60	1,46–1,75
Consumo de bebidas alcoólicas				
Não	1	-	1	-
Sim, mas sem risco de alcoolismo	1,04	0,83–1,31	1,22	0,98–1,52
Sim, mas com risco de alcoolismo	1,40	1,17–1,67	1,87	1,64–2,14
Tabagismo				
Não	1	-	1	-
Sim	1,01	0,86–1,18	2,26	1,98–2,57
Parição				
Primípara	1	-	1	-
1 filho	0,97	0,87–1,09	1,59	1,41–1,80
2 filhos	1,02	0,88–1,16	3,92	3,34–4,60
3 filhos ou mais	1,16	0,96–1,40	7,51	6,35–8,88
**Desfechos obstétricos adversos em gestações anteriores[b]**				
Óbito nenonatal				
Não	1		1	
Sim	0,61	0,46–0,81	0,61	0,48–0,76
Natimorto				
Não	1		1	
Sim	0,86	0,63–1,16	0,90	0,69–1,17

Prematuridade (IG < 37 semanas)				
Não	1		1	
Sim	1,05	0,80–1,29	1,44	1,23–1,69
Baixo peso ao nascer (< 2500g)				
Não	1		1	
Sim	0,95	0,80–1,14	1,37	1,18–1,59
Complicações maternas[c]				
Não	1		1	
Sim	0,95	0,81–1,12	1,22	1,04–1,42

IG = idade gestacional

([a]) Categoria de referência: Queria engravidar

[b]Excluídas as primíparas

[c]Complicacões em gestações anteriores (eclampsia, hipertensão arterial, rutura uterina ou diabetes)

Tabela 3 – Modelo multinomial final ajustado do planejamento da gestação. Nascer no Brasil, 2011/2012.

**Variáveis**	**Queria esperar mais([a])**		**Não queria engravidar([a])**	
	**OR**	**IC 95%**	**OR**	**IC 95%**
Idade (anos)				
< 20	1,89	1,68–2,14	2,42	2,10–2,79
20 - 34	0,43	0,37–0,51	0,75	0,64–0,88
≥ 35	1		1	
Anos de estudo				
≤ 7 anos	0,72	0,63–0,83	1,04	0,89–1,21
8 - 11 anos	0,99	0,87–1,12	1,38	1,21–1,58
≥12 anos	1		1	
Cor da pele				
Branca	1		1	
Preta	1,10	0,91–1,32	1,34	1,16–1,56
Parda	1,15	1,04–1,27	1,20	1,08–1,34
Amarela	1,56	1,05–2,32	1,39	0,93–2,08
Situação conjugal				
Com companheiro	1		1	
Sem companheiro	2,32	1,99–2,71	4,86	4,12–5,74
Trabalho remunerado				
Não	1		1	
Sim	1,15	1,04–1,27	1,14	1,04–1,25
Consumo de bebidas alcoólicas				
Não	1		1	
Sim, mas sem risco de alcoolismo	1,12	0,90–1,39	1,21	0,96–1,53
Sim, mas com risco de alcoolismo	1,25	1,04–1,50	1,36	1,17–1,58
Tabagismo				
Não	1			
Sim	0,87	0,73–1,04	1,23	1,06–1,43
Parição				
Primípara	1			
1 filho	1,30	1,16–1,46	2,49	2,15–2,88
2 filhos	1,55	1,34–1,80	6,96	5,81–8,33
3 filhos e mais	2,01	1,63–2,47	14,00	11,29–17,35
**Desfechos obstétricos adversos em gestações anteriores**[b]				
Óbito nenonatal				
Não	1		1	
Sim	0,61	0,44–0,85	0,44	0,34–0,57
Prematuridade (IG < 37 semanas)				
Não	1		1	
Sim	1,16	0,91–1,48	1,40	1,16–1,67
Baixo peso ao nascer (< 2500g)				

Não	1		1	
Sim	0,92	0,74–1,14	1,14	0,97–1,35
Complicações maternas[c]				
Não	1		1	
Sim	0,99	0,84–1,17	1,19	1,02–1,40

IG = idade gestacional

([a]) Categoria de referência: Queria engravidar

[b]Excluídas as primíparas

[c]Complicacões em gestações anteriores (eclampsia, hipertensão arterial, rutura uterina ou diabetes)
