# Supplementary material for: Factors associated with unintended pregnancy in Brazil: cross-sectional results from the Birth in Brazil National Survey, 2011/2012
Source: Reprod Health. 2016 Oct 17;13(Suppl 3):118. doi: 10.1186/s12978-016-0227-8 (PMC5073899; doi:10.1186/s12978-016-0227-8)
Supplement: Additional file 2: — Reviewer report. (PDF 168 kb) [file 12978_2016_227_MOESM2_ESM.pdf]

Referee's comments to the authors– this sheet WILL be seen by the author(s) and published with the article

Title	Factors associated with unintended pregnancy in Brazil: cross-sectional results from the Birth in Brazil National Survey, 2011/2012
Author(s)	Mariza Miranda Theme Filha, Marcia Leonardi Baldisserotto, Ana Claudia Santos Amaral Fraga, Susan Ayers, Silvana Granado Nogueira da Gama, Maria do Carmo Leal
Referee's name	Ana Langer

When assessing the work, please consider the following points, where applicable:

1. Is the question posed by the authors new and well defined?
2. Are the methods appropriate and well described, and are sufficient details provided to replicate the work?
3. Are the data sound and well controlled?
4. Does the manuscript adhere to the relevant standards for reporting and data deposition?
5. Are the discussion and conclusions well balanced and adequately supported by the data?
6. Do the title and abstract accurately convey what has been found?
7. Is the writing acceptable?

Please make your report as constructive and detailed as possible in your comments so that authors have the opportunity to overcome any serious deficiencies that you find and please also divide your comments into the following categories:

- Major Compulsory Revisions (which the author must respond to before a decision on publication can be reached)
- Minor Essential Revisions (such as missing labels on figures, or the wrong use of a term, which the author can be trusted to correct)
- Discretionary Revisions (which are recommendations for improvement but which the author can choose to ignore)

Where possible please supply references to substantiate your comments.

When referring to the manuscript please provide specific page and paragraph citations where appropriate.

General comments:

1. The paper addresses an important issue: unintended pregnancy and its risk factors. It also explores the effects of previous negative pregnancy outcomes on pregnancy intention, which is a new and important research question (see below).
2. The study, however, has some important weaknesses:
    a. The main or more innovative research question or hypothesis (page 5, 130-136) is that previous adverse pregnancy outcomes influence new pregnancy intention, but this hypothesis can only be explored in 60% of the sample, as 40% of the women are primiparous. All the other variables included in the model and described in the paper have well documented associations with pregnancy intention and this study only confirms previous findings in Brazil and elsewhere. I would focus this study and the paper on the main hypothesis of the research.
    b. Contraceptive use is an essential risk factor for pregnancy intention (for both timeliness and desire). The authors extensively talk about contraception and family planning in Brazil, but did not include contraceptive use (including type of method) in their model. I think this is a big omission that should be corrected or the rationale for not including contraceptive use explained in full detail.

(continue on the next sheet)

Continued:

c. The authors use in a non-systematic way the terms unintended, mistimed and unwanted pregnancy (e.g., on page 6, line 159 it should say "….whether their pregnancy was intended" instead of "wanted"). Considering the important distinctions between these terms in general and for this paper, the authors should make sure that they are used correctly.

d. The authors say that unwanted and mistimed pregnancies are a health problem (page 3, line 87) but don't explain their consequences on women and children's health, human development and wellbeing. Adding text and references on that would make the paper stronger.

e. Some associations are statistically significant, but the reason why that is the case is not immediately apparent to the reader and should be explained by the authors, e.g., why would smoking represent a risk of mistimed or unwanted pregnancy? Other associations are so obvious that finding again that women with no partner, no paid job or three or more children are at high risk of mistimed or unwanted pregnancy does not add much to the existing literature or understanding of factors associated with unintended pregnancy.

f. The discussion section does not include any reflection on policy and programmatic implications of the study findings, or recommendations.

g. The abstract is not clear, in particular the methods section, where the variables are not described. Also in the abstract, some sentences are difficult to interpret, i.e., lines 71-72 ("…and the chances of complication…..respectively"). Lines 73-74 would read better if the authors said that previous neonatal death had a protective effect on mistimed and unwanted pregnancy, instead of how it's phrased now.

Major compulsory revisions:

a. Limit the analysis and paper to the main hypothesis of the study, i.e. effects of adverse pregnancy outcomes on pregnancy intention. This means that only women with previous pregnancies should be included in the study sample.
b. Add contraceptive use to the list of explored variables. Understanding the effects contraceptive use and type of method on unwanted and mistimed pregnancy would allow the authors to make much more practical and relevant recommendations. If there was a rationale for not including this variable in the study and model, it should be explained clearly and convincingly.
c. Discuss policy and programmatic implications of the findings and make recommendations for future research and action.
d. Improve the English and/or writing, as sometimes the sentences are not clear.

Minor essential revisions:

a. When the authors discuss the mechanisms through which a previous preterm birth may affect unintended pregnancy, they do not make a reference to an obvious one: the possibility of the previous child having important health complications (page 13, lines 310-313)., which is likely to discourage a new pregnancy.
b. There is a discussion on postpartum depression that is not necessary, considering that this problem is not mentioned in any other sections of the paper (page 13, 316-321)

Discretionary revisions:

Referee's comments to the authors– this sheet WILL be seen by the author(s) and published with the article

Title	Factors associated with unintended pregnancy in Brazil: cross-sectional results from the Birth in Brazil National Survey, 2011/2012
Author(s)	Mariza Miranda Theme Filha, Marcia Leonardi Baldisserotto, Ana Claudia Santos Amaral Fraga, Susan Ayers, Silvana Granado Nogueira da Gama, Maria do Carmo Leal
Referee's name	José Guilherme Cecatti

When assessing the work, please consider the following points, where applicable:

1. Is the question posed by the authors new and well defined?
2. Are the methods appropriate and well described, and are sufficient details provided to replicate the work?
3. Are the data sound and well controlled?
4. Does the manuscript adhere to the relevant standards for reporting and data deposition?
5. Are the discussion and conclusions well balanced and adequately supported by the data?
6. Do the title and abstract accurately convey what has been found?
7. Is the writing acceptable?

Please make your report as constructive and detailed as possible in your comments so that authors have the opportunity to overcome any serious deficiencies that you find and please also divide your comments into the following categories:

- Major Compulsory Revisions (which the author must respond to before a decision on publication can be reached)
- Minor Essential Revisions (such as missing labels on figures, or the wrong use of a term, which the author can be trusted to correct)
- Discretionary Revisions (which are recommendations for improvement but which the author can choose to ignore)

Where possible please supply references to substantiate your comments.

When referring to the manuscript please provide specific page and paragraph citations where appropriate.

General comments: This is a really interesting, well performed study and well written manuscript exploring new insights on the risk factors for unintended pregnancy in Brazil, with special emphasis on previous adverse obstetric outcomes as predictors. Que question is well defined, the methods are appropriate, exploring a huge database with information representative of all women delivering in the country and sufficiently detailed. Data analysis were well described and properly performed. The results are sound and properly discussed.

Major compulsory revisions:

Minor essential revisions:
In the abstract, I suggest to specify the outcomes used (mistimed or unwanted pregnancy) in the methods sub item. In the introduction session, let me recommend authors see and perhaps include a reference on the social and public health impact of unintended pregnancies in Brazil (Le HH et al., Int J Womens Health 2014). Still in the introduction, lines 121-125, it is mentioned the negative maternal and child health outcomes from unintended pregnancies, what is not the focus of the current study. I suggest to delete that.
In the methods session, please provide a more detailed information regarding the original study, including some information on data collection and management, control of quality, etc. Although I

(continue on the next sheet)

Continued:

understand that such information is already available in other publications, this would improve the self-understanding of this article.
No mention at all to the STROBE statement or checklist is done.
For lines 174-176, an adequate reference should be provided.
In the discussion session, some results are extensively provided, like in lines 287-290. This is not an appropriate place to report such results. The same should be consistently checked through the whole text.
Reference number 6 and 28 are the same.
Table 1: I suggest to include the missing values for each variable in footnote

Discretionary revisions:
